# Sexual partnership age pairings and risk of HIV acquisition in rural South Africa

**DOI:** 10.1097/QAD.0000000000001553

**Published:** 2017-07-12

**Authors:** Adam Akullian, Anna Bershteyn, Daniel Klein, Alain Vandormael, Till Bärnighausen, Frank Tanser

**Affiliations:** a Institute for Disease Modeling, Global Good Fund, Bellevue, Washington, USA; b Africa Health Research Institute, University of KwaZulu-Natal, Mtubatuba, South Africa; c Institute for Public Health, Faculty of Medicine, University of Heidelberg, Heidelberg, Germany; d Department of Infection and Population Health, Institute of Epidemiology and Health Care, University College London, London, UK.

**Keywords:** age gaps, HIV transmission, sexual behavior, South Africa

## Abstract

**Objective:**

To quantify the contribution of specific sexual partner age groups to the risk of HIV acquisition in men and women in a hyperendemic region of South Africa.

**Design:**

We conducted a population-based cohort study among women (15–49 years of age) and men (15–55 years of age) between 2004 and 2015 in KwaZulu-Natal, South Africa.

**Methods:**

Generalized additive models were used to estimate smoothed HIV incidence rates across partnership age pairings in men and women. Cox proportional hazards regression was used to estimate the relative risk of HIV acquisition by partner age group.

**Results:**

A total of 882 HIV seroconversions were observed in 15 935 person-years for women, incidence rate = 5.5 per 100 person-years [95% confidence interval (CI), 5.2–5.9] and 270 HIV seroconversions were observed in 9372 person-years for men, incidence rate = 2.9 per 100 person-years (95% CI, 2.6–3.2). HIV incidence was highest among 15–24-year-old women reporting partnerships with 30–34-year-old men, incidence rate = 9.7 per 100 person-years (95% CI, 7.2–13.1). Risk of HIV acquisition in women was associated with male partners aged 25–29 years (adjusted hazard ratio; aHR = 1.44, 95% CI, 1.02–2.04) and 30–34 years (aHR = 1.50, 95% CI, 1.08–2.09) relative to male partners aged 35 and above. Risk of HIV acquisition in men was associated with 25–29-year-old (aHR = 1.72, 95% CI, 1.02–2.90) and 30–34-year-old women (aHR = 2.12, 95% CI, 1.03–4.39) compared to partnerships with women aged 15–19 years.

**Conclusion:**

Age of sexual partner is a major risk factor for HIV acquisition in both men and women, independent of one's own age. Partner age pairings play a critical role in driving the cycle of HIV transmission.

## Introduction

Recent empirical evidence from South Africa has challenged the hypothesis that older male sexual partners significantly increase the risk of HIV transmission to young women [1–3], and previous research from East Africa has been inconsistent as to whether large age gaps are associated with the risk of HIV acquisition in women [4,5]. Though the age-dependent structure of sexual partnerships in Sub-Saharan Africa [6–8] tends to place young women at higher risk of HIV acquisition than young men [9], large age gaps may not be a key driver of the epidemic [10]. These recent studies call into question the effectiveness of educational campaigns that aim to discourage sexual relations between young women and substantially older men.

Although the relative difference in age between partners may not be associated with HIV acquisition, certain high-risk age pairings may still play a role in driving the epidemic for both men and women. It was previously hypothesized that older men (ages 35 and above) posed a disproportionate risk of HIV transmission to younger women (aged < 25) [11–14] because of their higher HIV prevalence and lower rates of condom use [15,16], though these observations were based on cross-sectional or ecological data [17]. Women in age-disparate relationships, furthermore, have less control over practicing safer sex [11,15,18,19].

In high burden areas of sub-Saharan Africa, however, younger men (<35 years of age) are more likely to engage in risky sexual behavior, including multiple partners [20] and carry the highest risk of becoming newly infected with HIV [21]. Younger men are also less likely to be aware of their HIV status [22], and those infected with HIV are less likely to be on antiretroviral therapy (ART) [23] and more likely to have detectable viral load [24]. Whether men in this age group pose a greater risk of onward transmission to their partners has not been rigorously tested. Empirical studies linking male partner age to new HIV infections in women are thus needed to better identify high-risk partnerships for targeted HIV prevention, as well as to understand the underlying biological and behavioral drivers of risk. Similarly, epidemiologic data on which female age groups contribute to the highest risk of HIV acquisition in men are lacking and will be crucial to inform HIV prevention in men.

In this study, we move beyond relative age gaps to address the contribution of specific sexual partner age groups to HIV risk in both men and women in a hyperendemic region of South Africa. We test for the independent, nonlinear effects of self-reported partner age on risk of HIV acquisition in men and women. Our results provide empirical evidence that can be used to identify both high-risk age groups and high-risk partnership age pairings, and provide biological and behavioral insight into the age-specific cycle of HIV transmission in high-burden settings.

## Methods

### Study design

We conducted a population-based cohort study using data collected between 2004 and 2015 from a longitudinal surveillance system conducted by the Africa Health Research Institute's demographic surveillance information system in a rural area of KwaZulu-Natal, South Africa. A detailed description of the study population and data collection methods have been described previously [25]. In brief, the study area is located near the town of Mtubatuba in the Umkanyakude district of KwaZulu-Natal, and has approximately 85 000 residents of all ages who are members of about 11 000 households [25]. The area is characterized by high unemployment – upwards of 80% – and high rates of cyclical migration, in which individuals temporarily change residences for labor-related activities [26,27]. HIV prevalence is high (>50% among men over 30 and women over 25 years of age), and though HIV incidence has begun to decline in recent years, it remains high [28]. Rapid scale-up of ART began in 2004 and ART coverage rose to 30% in 2011 among HIV-positive adults, with large discrepancies in coverage by age and sex (ART coverage rates in year 2011: 15% among women 15–24, 40% among women 25–49, 8.5% among men 15–24, 30% among men 25–49) [23]. Africa Centre Demographic Information System study procedures were approved by the University of KwaZulu-Natal biomedical research ethics committee.

Inclusion into the study was restricted to women 15–49 years of age (prior to 2007, HIV testing was only offered to women under age 50) and men 15–55 years of age, with at least one HIV-negative test followed by at least one HIV test (positive or negative). Participants were censored at age 50 for women and 56 for men. Participants were restricted to those who responded to at least one annual general health interview (described below) occurring no earlier than 12 months before first HIV-negative test and no later than 12 months after last HIV test. Seroconversion date was simulated in a single trial as a random date between last HIV negative and first HIV-positive test, following methods from previous studies in the same cohort [27,29]. Respondents who met all inclusion criteria but did not provide the age of their most recent sexual partner were separately analyzed to examine potential biases. HIV prevalence was also estimated for reference, and included individuals with at least one HIV test between 2004 and 2015.

### Exposure and outcome measurement

**Fig. 1:**
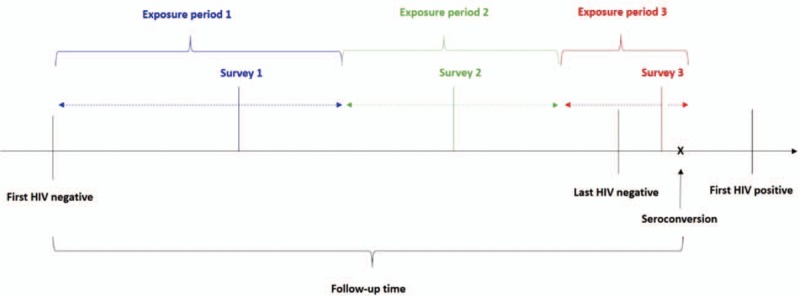
Person-time between study entry (first HIV-negative test) and exit (seroconversion or lost to follow-up) is broken into exposure periods that span from study entry to midway between survey dates (for the first exposure period) and from midway between survey dates to study exit (for the last exposure period).

The annual data collection cycle included HIV testing by dried blood spots as well as face-to-face general health interviews administered to all eligible and consenting adults by field workers from the local community to ascertain sexual risk behavior. The primary outcome was time to HIV acquisition as measured by positive HIV serostatus by antibody testing with two rapid tests. Individual uptake of HIV testing is approximately 80%. The primary exposure of interest was reported age of most recent sexual partner. Person-time between first HIV-negative test (observation start) and last HIV test (observation end) was divided into intervals of follow-up time corresponding to the nearest interview date (Fig. 1). For the primary analysis, person-time was only allocated to follow-up time where information on partner age was not missing.

The start date of the first exposure period was assigned to the later of observation start or one year prior to first interview. The exposure period end date for the last interview was the earliest of observation end or one-year following last interview.

### Statistical analysis

Person-time incidence rates and 95% confidence intervals (CI) were calculated for potential risk factors of HIV infection, stratified on age groups (15–24 and 25–49 years for women and 15–24, 25–34, and 35–55 years for men). Potential risk factors included frequency of condom use with most recent partner [always, sometimes (defined as those who reported having ever used a condom with their most recent partner but not using a condom every time), and never]; number of sexual partners in the previous 12 months (0, 1, 2 and so on); whether the male respondents were circumcized (yes/no); and the reported age of the most recent sexual partner (15–19, 20–24, 25–29, 30–34, and 35 and above years of age).

We explored nonlinear trends in risk by fitting Poisson generalized additive models (GAM) to estimate smoothed HIV incidence rates across all combinations of respondent age and reported partner age. We fit separate GAMs for both men and women using a locally weighted regression smoother and an offset for person-time at risk [30], as follows: 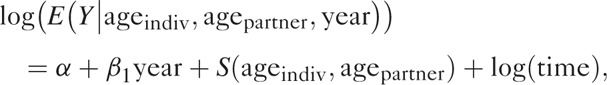


where  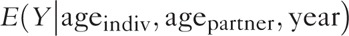 is the expected number of seroconversions given an individual's age, reported partner's age, and year of observation;  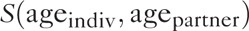 is a locally weighted smoother (loess) across all partnership age pairings; and log(time) is an offset for follow-up time expressed in days. Next, fitted incidence rates were estimated at the median year, 2009, and plotted on a prediction grid of pairwise partnership age pairings. Credible intervals were then generated by randomly permuting the outcome (HIV seroconversion) 1000 times across all fixed partnership age pairs (keeping the overall seroconversion rate constant) and fitting the GAM after each permutation. The 99% upper credible bound was then overlaid on the prediction surface, delineating the area of parameter space in which HIV incidence is greater than the overall incidence without an age-pairing effect. Significance levels were set at α (type I error rate) less than 0.01 to account for multiple testing in the permutation tests [31].

The effect of partner age on HIV risk was evaluated in multivariate Cox proportional hazards regression models, adjusting for the potentially confounding effects of individual risk factors, including respondent age, frequency of self-reported condom use, and reported number of sexual partners in previous year. Adjustment for these factors was done to remove the effects of known individual-level risk factors on HIV risk that may also be associated with pairing with a specific age group. Year of observation was adjusted for in all models. Multivariate regression models were run using complete case analysis, which drops any record with missing information on any of the covariates in the model. The proportionate hazards assumptions of each covariate, and of the model as a whole, were tested using both Schoenfeld and scaled Schoenfeld residuals. All statistical analyses were done using STATA 14 (STATA Corp, College Station, Texas, USA) and the R statistical language version 3.2.2, using the mgcv package [32,33].

## Results

Included in the full cohort (regardless of whether age of recent sexual partner was reported) were 10 260 women (40 906 person-years of observation) and 7839 men (28 645 person-years) (Suppl Figure 1). Between 2004 and 2015, 1788 new HIV infections were recorded in women, [incidence rate per 100 person-years (incidence rate) = 4.4, 95% CI, 4.2–4.6], and 579 new HIV infections were recorded in men, (incidence rate = 2.0, 95% CI, 1.9–2.2). The age-specific incidence rate in the full sample was greatest in women 20–24 years of age, (incidence rate = 6.9, 95% CI, 6.4–7.4) and in men 25–29 years of age, (incidence rate = 3.9, 95% CI, 3.3–4.7; Figure 1).

**Fig. 2:**
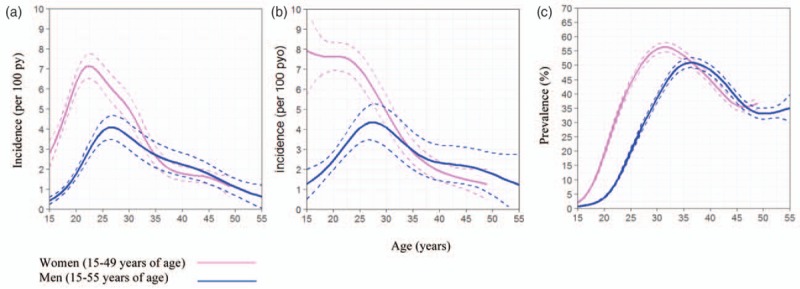
Age-specific HIV incidence with 95% confidence intervals for men and women between 2004 and 2015 for the full dataset (including individuals who had not initiated sex) (a); restricted to those who reported the age of their most recent sexual partner (b) and age-specific HIV prevalence with 95% confidence interval for men and women between 2004 and 2015 (c).

Those with nonmissing information on age of most recent sexual partner included 7251 (59.3%) women and 4973 (40.7%) men. In this group, 882 new HIV infections were observed in 15 935 person-years for women, (incidence rate = 5.5, 95% CI, 5.2–5.9) and 270 new HIV infections were observed in 9372 person-years for men, (incidence rate = 2.9, 95% CI, 2.6–3.2). In this group, incidence peaked in women 15–24 years of age (incidence rate = 7.7, 95% CI 7.1–8.3) and peaked in men 25–29 years of age (incidence rate = 4.2, 95% CI 3.3–5.4). HIV prevalence (estimated at the median year, 2009) peaked (upwards of 50%) in women between age 27 and 35 years and in men between 32 and 40 years (Fig. 2).

**Fig. 3:**
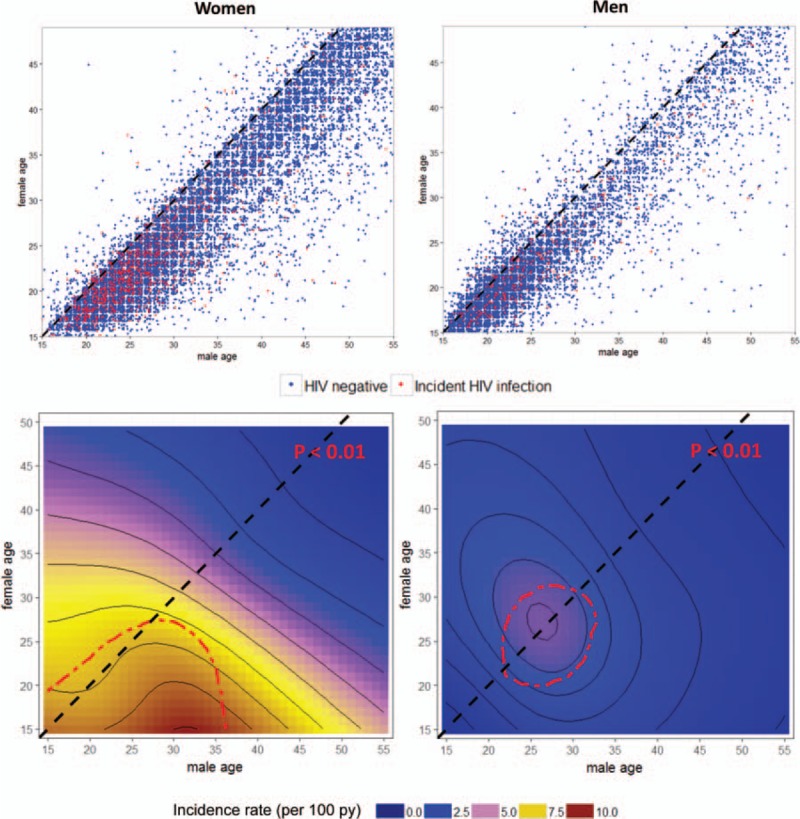
(Top panel) scatterplot displaying all reported relationship age pairings and associated incident HIV infections for men and women.

Crude, age-specific incidence rates by selected risk factors, including partner age, are shown in Table 1. The highest incidence was in women 15–24 years of age who reported a recent partner's age of 30–34 years, (incidence rate = 9.7, 95% CI, 7.2–13.1) followed by 25–29 years, (incidence rate = 8.2, 95% CI, 7.2–9.4). Women 25–49 who reported a partner's age of 25–29 years were also at high risk, (incidence rate = 7.0, 95% CI, 5.6–8.8). Scatter plots showing the distribution of all reported partnership age pairings, coded by whether they resulted in a seroconversion or not, are shown in Fig. 3. We observed significantly higher risk of HIV acquisition among women under 25 years of age who reported recent partnerships with men up to 35 years of age (*P* < 0.01 Figure 3). In men, the highest risk of HIV acquisition was in those 25–30 years who reported recent partnerships with similarly age women, (*P* < 0.01).

(Bottom panel) smoothed incidence rates of HIV by reported age pairings for men and women. Greater than expected incidence – compared to the mean incidence with no age-pair effect – is depicted within the red-dashed upper 99% credible bounds, generated by iterating the smoothed model under the null hypothesis of no age-pair effect.

HIV risk in women was associated with younger male partner age (<35 years) in both crude and adjusted models (Table 2). In models adjusted only for year of observation, women who reported partnerships with men under 35 years of age experienced a more than three-fold increased risk of HIV compared with those who reported partnerships with men 35 and above years of age (*P* < 0.001). In models additionally adjusted for the respondent's age, HIV incidence in women was associated with male partners aged 25–29 years [adjusted hazard ratio; aHR = 1.44, 95% CI, 1.02–2.04] and 30–34 years (aHR = 1.50, 95% CI, 1.08–2.09) relative to male partners aged 35 and above years. In models additionally adjusted for individual-level sexual behavior [frequency of self-reported condom use of the respondent (never, sometimes, and always), and reported number of sexual partners in previous year (0, 1, 2 and so on)], women who reported partnerships with men 30–34 years had significantly higher risk of HIV compared with those who reported male partners 35 and above years (aHR = 1.46, 95% CI, 1.05–2.04). HIV risk in men was significantly associated with reported female partner age in both crude and adjusted models. In multivariate adjusted models, HIV risk was significantly elevated among men who reported female partners 25–29 years of age (aHR = 1.78, 95% CI, 1.06–2.99) and 30–34 years of age (aHR = 2.23, 95% CI, 1.08–4.61) compared with men who reported partnerships with women in the youngest age group (15–19 years). All proportionate hazards assumptions held based on tests of Schoenfeld residuals at α less than 0.05.

## Discussion

In this population-based cohort study, we found evidence of nonlinear associations between reported partner age and risk of HIV acquisition in men and women. HIV incidence was elevated in women who reported recent sexual partnerships with men 25–34 years of age. In contrast to previously held beliefs that older men drive HIV risk in women, report of male age 35 and over was not associated with elevated risk of HIV acquisition in women. The highest risk of HIV acquisition for men was among those who reported partnerships with women 25–34 years of age. These results were robust to adjustment for individual-level sexual risk behaviors of those at risk of HIV acquisition.

Our study provides more insight into why previous research from South Africa either failed to find an association between age gap and risk of HIV in young women [1,2] or found lower risk of HIV with increasing age gap in older women [34]. Age gaps may only increase the risk of HIV acquisition in women to the extent that they involve men in high-risk age groups. The higher risk that men 25–34 years of age pose to women was also observed in older women (25–49 years of age Suppl Table 1), indicating that this relationship is driven less by relative age gap between partners and more by factors associated with the age group of men 25–34 years. The risk of HIV acquisition in men was highest among those who reported recent sexual activity with women 25–34 years of age, corresponding to the age of peak prevalence in women, which occurs 5–10 years following peak incidence in women. The general cycle of transmission ensues: men 25–34 years of age transmit recent HIV infection to younger women 15–24 years of age, who then age into a group at greatest risk of transmission to their similar-aged male partners. This cycle of transmission, albeit an oversimplified version of the true dynamics of HIV transmission, is consistent with the age structure of partnerships in this population [7] and provides key insights into potential courses of intervention.

Three key mechanisms can explain the efficiency of HIV transmission from men 25–34 to their younger female partners. First, though men in this age group are not yet at peak prevalence, they experience the highest rate of HIV acquisition. These individuals have disproportionately acute and early (primary) HIV infections, have low ART coverage (<10% in 2011 [29]), and high loss to follow-up in the cascade of care [35]. In addition to the increased viral load characterized by recent HIV infection [36], newly infected individuals are less likely to be aware of their HIV status and may be in a period of life where high-risk behavior is common, including multiple partnerships, lower condom use, and high coital frequency [37]. Our findings are biologically plausible considering the high per sexual act transmission probability with individuals with high viral load [38,39], and are behaviorally plausible given that the same behaviors that lead to acquisition are also associated with onward transmission [37]. Although acutely infected young women are also at high risk of transmitting to their HIV-negative male partners, the structure of the sexual network (in which men tend to partner with women their age or younger), results in a delay of high-risk transmission of 5–10 years.

Our results are consistent with recent phylogenetic analyses from KwaZulu-Natal, South Africa that found close genetic linkages between young HIV-positive women (aged 15–24) and HIV-positive men in their 30's (average age of 31.5 years), as well as evidence linking HIV infection in men to women of the same age group [40]. The phylogenetic results also support our hypothesis that the men who pose the greatest risk of transmitting HIV to young women are those with undiagnosed or unsuppressed HIV infection, characteristic of early and acute HIV infection. In the study, among the 79 HIV-positive men with viral load more than 1000 copies/ml who were linked to HIV infection in women under 40, 78.5% were unaware of their HIV-positive status, 96.2% were not on ART, and 36.7% had viral loads more than 50 000 copies/ml [40]. HIV-positive men with genetic linkages to recently infected women less than 25 years of age, furthermore, were found to have high median viral load of more than 200 000 copies/ml [41], further supporting the hypothesis that acutely infected men are high-risk transmitters to young women. Phylogenetic studies on HIV transmission, however, are subject to inherent limitations that epidemiologically based data analyses can overcome. Our study, for example employed a much larger sample size, included potential exposures to partners outside of the study population, and included age groups less represented in phylogenetic studies (e.g. those with viral load cutoff values <1000 copies/ml). Considering these differences, the consistency of our study results with recent phylogenetic analyses further supports the hypothesized cycle of transmission observed in both analyses.

Despite the overwhelming evidence that certain partner age groups contributed more to risk of HIV acquisition in women than others, it is important to note that incidence in sexually active young women was high across all male partner age groups (ranging from 7.2 to 9.7 per 100 person-years). Our results should thus not overshadow the alarmingly high overall HIV incidence in young women regardless of age of male sexual partner. Furthermore, although acute and early infections account for a disproportionate number of new transmissions in concentrated or early-stage epidemics [42,43], their role in sustaining HIV transmission in generalized or hyperepidemic settings is unclear [44–46]. Notwithstanding these uncertainties, the interaction between recent HIV infection, low ART coverage, and risky sexual behavior in key age groups is likely to contribute substantially to onward transmission. Further study can clarify the relative contribution of each component.

Our study contributes to a growing body of evidence suggesting that the risk profile of potential partners, often approximated by community-level measures of viral load, ART coverage, and risky sexual behavior, plays a major role in driving HIV risk [28,29,36,47]. Partner age may be a useful proxy for a suite of biological and behavior risk factors hypothesized to increase the risk of onward transmission. Mathematical modeling can use empirical data to update previous estimates on the effect that age-specific interventions might have on preventing new HIV infections [6]. Our results may help to further clarify model-based uncertainties around the population-level benefit of treatment as prevention, preexposure prophylaxis, voluntary male medical circumcision, behavior change campaigns, and other modes of HIV prevention.

Our study has some limitations. First, the use of self-reported sexual risk behavior data is subject to both selection bias and reporting bias. We relied on individuals to accurately report on the age of their most recent sexual partner. Previous studies indicate that men and women may selectively report certain partnerships over others [48]. Though we expect some degree of partnership age misreporting, this error is not likely to be systematically biased [49]. Any failure to report certain partner types or misrepresentation of partner age will tend to shift the effect estimates toward the null: resulting in a conservative bias. Second, we did not have partner-specific biologic or behavioral data available and our ability to link transmission events to the transmitting partner is limited by the accuracy of the estimated seroconversion date. Finally, we did not have complete data on age of recent sexual partner. Women 15–24 with missing information on partner age tended to having a higher incidence than those who reported their partner's age (8.3 versus 7.7 per 100 person-years), suggesting some degree of selection bias.

Our results highlight the important role of specific age groups in driving the cycle of HIV transmission in hyperendemic regions, especially in areas with similar behavioral epidemiology as KwaZulu-Natal. Whether these results are generalizable across epidemic settings is unclear, and further studies using similar methods will be needed to validate these results, taking into consideration the contextual effects of region-specific sexual networks, demography, and HIV epidemiology [50]. Notwithstanding differences in effect across region, our results shed new light on the potentially limited role of large age gaps in driving incidence in young women. Positive prevention efforts should focus not only on age groups at high risk of HIV acquisition, but also on age groups with high potential for onward transmission.

## Acknowledgements

The authors would like to thank the Africa Health Research Institute (AHRI) staff and all those involved in data collection conducted by the Africa Centre Demographic Information System (ACDIS). This project would not have been possible without the participation of the research participants, who contributed time, survey responses, and biological samples for this study.

A.A. conceived of the study and conducted the analysis and wrote the paper. A.B. and D.K. provided critical analytical contributions including revision of the manuscript for important intellectual content. A.V. contributed to acquisition of the data and analytical methods, T.B. and F.T. contributed to critical revision of the manuscript for important intellectual content.

A.A., A.B., and D.K. were supported by Bill and Melinda Gates through the Global Good Fund. F.T. and A.V. were supported by the South African Medical Research Council (SA MRC) Flagship grant (MRC-RFA-UFSP-01-2013/UKZN HIVEPI). F.T. was supported by two National Institutes of Health grants (R01HD084233 and R01AI124389) and a UK Academy of Medical Sciences Newton Advanced Fellowship (NA150161). T.B. was supported by the Alexander von Humboldt Foundation funded by the German Federal Ministry of Education and Research, the Wellcome Trust, the European Commission, the Clinton Health Access Initiative, and the National Institutes of Health Fogarty International Center (D43-TW009775). Funding for the Africa Health Research Institute's Demographic Surveillance Information System and Population-based HIV Survey was received from the Wellcome Trust. The funders had no role in study design, data collection and analysis, decision to publish, or preparation of the manuscript.

### Conflicts of interest

There are no conflicts of interest.

## Supplementary Material

Supplemental Digital Content

## Figures and Tables

**Table 1:** Age-specific incidence rates (incidence rate) of HIV by selected self-reported risk factors.

	Women								Men											
	15–24				25–49				15–24				25–34				35–55			
Age group	Person-years	New infections	IR	95% CI	Person-years	New infections	IR	95% CI	Person-years	New infections	IR	95% CI	Person-years	New infections	IR	95% CI	Person-years	New infections	IR	95% CI
Full sample	21043	1173	5.6	(5.3–5.9)	19863	615	3.1	(2.9–3.4)	17915	296	1.7	(1.5–1.9)	5061	184	3.6	(3.1–4.2)	5668	99	1.7	(1.4–2.1)
Ever had sex																				
Yes	11306	891	7.9	(7.4–8.4)	18063	565	3.1	(2.9–3.4)	3407	84	2.5	(2.0–3.1)	1919	69	3.6	(2.8–4.6)	1266	18	1.4	(0.9–2.3)
No	7129	156	2.2	(1.9–2.6)	255	3	1.2	(0.4–3.6)	4755	26	0.5	(0.4–0.8)	277	2	0.7	(0.2–2.9)	119	2	1.7	(0.4–6.7)
Missing^a^	2608	126	4.8	(4.1–5.8)	1545	47	3.0	(2.3–4.0)	9753	186	1.9	(1.7–2.2)	2865	113	3.9	(3.3–4.7)	4283	79	1.8	(1.5–2.3)
Reported partner age	7782	599	7.7	(7.1–8.3)	8153	283	3.5	(3.1–3.9)	5450	145	2.7	(2.3–3.1)	2231	90	4.0	(3.3–5.0)	1690	35	2.1	(1.5–2.9)
Missing partner age^b^	3531	292	8.3	(7.4–9.3)	9917	283	2.9	(2.5–3.2)	1067	27	2.5	(1.7–3.7)	730	29	4.0	(2.7–5.7)	710	10	1.4	(0.8–2.6)
Frequency of condom use with most recent partner^b^																				
Always	2103	181	8.6	(7.4–10.0)	928	56	6.0	(4.6–7.8)	2367	49	2.1	(1.6–2.7)	556	22	4.0	(2.6–6.0)	129	3	2.3	(0.7–7.2)
Sometimes	2857	216	7.6	(6.6–8.6)	2100	109	5.2	(4.3–6.3)	1726	44	2.5	(1.9–3.4)	835	39	4.7	(3.4–6.4)	322	13	4.0	(2.3–7.0)
Never	3393	249	7.3	(6.5–8.3)	6745	155	2.3	(2.0–2.7)	1749	63	3.6	(2.8–4.6)	1027	37	3.6	(2.6–5.0)	1644	24	1.5	(1.0–2.2)
Missing	2963	245	8.3	(7.3–9.4)	8297	246	3.0	(2.6–3.4)	942	25	2.7	(1.8–3.9)	653	26	4.0	(2.7–5.8)	607	9	1.5	(0.8–2.9)
Sex partners in past 12 months^b^																				
0	303	15	4.9	(3.0–8.2)	1111	23	2.1	(1.4–3.1)	104	1	1.0	(0.1–6.8)	65	0	0.0	NA	79	1	1.3	(0.2–9.0)
1	7925	617	7.8	(7.2–8.4)	8620	289	3.4	(3.0–3.8)	2004	43	2.1	(1.6–2.9)	1030	36	3.5	(2.5–4.8)	551	7	1.3	(0.6–2.7)
2 and so on	125	13	10.4	(6.0–17.9)	47	8	16.9	(8.5–33.8)	358	14	3.9	(2.3–6.6)	167	7	4.2	(2.0–8.8)	31	1	3.2	(0.5–23.0)
Missing	2953	246	8.3	(7.4–9.4)	8284	245	3.0	(2.6–3.4)	942	26	2.8	(1.9–4.1)	656	26	4.0	(2.7–5.8)	606	9	1.5	(0.8–2.9)
Age of most recent partner																				
15–19	777	57	7.3	(5.7–9.5)	3	0	0	NA	3436	78	2.3	(1.8–2.8)	113	1	0.9	(0.1–6.3)	5	0	0.0	NA
20–24	3961	286	7.2	(6.4–8.1)	36	1	2.8	(0.4–19.8)	1919	63	3.3	(2.6–4.2)	988	40	4.1	(3.0–5.5)	33	1	3.1	(0.4–21.8)
25–29	2472	203	8.2	(7.2–9.4)	1061	74	7.0	(5.6–8.8)	93	3	3.2	(1.0–10.0)	832	36	4.3	(3.1–6.0)	145	4	2.8	(1.0–7.3)
30–34	434	42	9.7	(7.2–13.1)	1516	89	5.9	(4.8–7.2)	3	1	38.9	(5.5–276.0)	259	13	5.0	(2.9–8.6)	318	7	2.2	(1.1–4.6)
35 and above	138	11	7.9	(4.4–14.3)	5538	119	2.1	(1.8–2.6)	1	0	0.0	NA	40	0	0.0	NA	1190	23	1.9	(1.3–2.9)
Circumcized																				
Yes	NA	NA	NA	NA	NA	NA	NA	NA	821	7	0.9	(0.4–1.8)	256	7	2.7	(1.3–5.7)	122	1	0.8	(0.1–5.8)
No	NA	NA	NA	NA	NA	NA	NA	NA	5796	87	1.5	(1.2–1.9)	1833	65	3.5	(2.8–4.5)	1633	21	1.3	(0.8–2.0)

Person time in years. CI, confidence interval; IR, crude incidence rate per 100 person-years; NA, not applicable.

^a^Missing information on survey question “Have you ever had sex?” with unknown reason for missingness.

^b^Among those who answered “yes” to survey question “Have you ever had sex?”

**Table 2:** Hazard ratios for the association between select risk factors and incident HIV infections.

	Women (ages 15–49)									Men (ages 15–55)								
	Year adjusted			Year and age adjusted			Multivariate adjusted			Year adjusted			Year and age adjusted			Multivariate adjusted		
	aHR	95% CI	*P* value	aHR	95% CI	*P* value	aHR^a^	95% CI	*P* value	aHR	95% CI	*P* value	aHR	95% CI	*P* value	aHR^a^	95% CI	*P* value
Age of respondent																		
15–19	2.56	(2.17–3.01)	<0.001				3.14	(2.00–4.92)	<0.001	0.49	(0.37–0.65)	<0.001				1.42	(0.61–3.29)	0.418
20–24	4.01	(3.43–4.69)	<0.001				3.22	(2.12–4.87)	<0.001	1.54	(1.21–1.95)	<0.001				1.99	(0.94–4.22)	0.072
25–29	3.40	(2.84–4.08)	<0.001				2.48	(1.66–3.69)	<0.001	2.36	(1.82–3.07)	<0.001				2.27	(1.12–4.59)	0.022
30–34	2.42	(1.94–3.01)	<0.001				1.92	(1.33–2.79)	0.001	1.83	(1.32–2.55)	<0.001				1.69	(0.88–3.26)	0.114
35–55	1.00	Ref					1.00	Ref		1.00	Ref					1.00	Ref	
Frequency of condom use with most recent partner																								
Never	1.00	Ref		1.00	Ref		1.00	Ref		1.00	Ref		1.00	Ref		1.00	Ref	
Sometime	1.64	(1.42–1.91)	<0.001	1.21	(1.04–1.41)	0.013	1.23	(1.04–1.44)	0.013	1.29	(0.98–1.70)	0.067	1.16	(0.87–1.53)	0.315	1.13	(0.84–1.51)	0.430
Always	1.96	(1.66–2.30)	<0.001	1.36	(1.15–1.61)	<0.001	1.41	(1.19–1.68)	<0.001	0.92	(0.68–1.23)	0.569	0.87	(0.64–1.18)	0.372	0.86	(0.63–1.19)	0.374
Sex partners in past 12 months																		
0	1.00	Ref		1.00	Ref		1.00	Ref		1.00	Ref		1.00	Ref		1.00	Ref	
1	2.02	(1.46–2.80)	<0.001	1.37	(0.98–1.90)	0.062	1.28	(0.89–1.84)	0.179	2.16	(1.11–4.21)	0.024	2.11	(1.08–4.11)	0.029	1.69	(0.83–3.43)	0.148
2+	4.61	(2.70–7.87)	<0.001	2.50	(1.46–4.27)	0.001	1.93	(1.05–3.55)	0.034	2.91	(1.45–5.84)	0.003	2.66	(1.32–5.36)	0.006	2.15	(1.02–4.53)	0.043
Age of most recent partner																		
15–19	3.18	(2.33–4.34)	<0.001	1.23	(0.77–1.95)	0.379	1.16	(0.73–1.85)	0.529	1.00	Ref		1.00	Ref		1.00	Ref	
20–24	3.15	(2.56–3.88)	<0.001	1.21	(0.84–1.76)	0.310	1.15	(0.79–1.67)	0.464	1.70	(1.27–2.29)	<0.001	1.39	(0.97–1.98)	0.072	1.43	(1.00–2.04)	0.052
25–29	3.50	(2.84–4.32)	<0.001	1.44	(1.02–2.04)	0.040	1.38	(0.97–1.95)	0.073	1.97	(1.36–2.86)	<0.001	1.72	(1.02–2.90)	0.041	1.78	(1.06–2.99)	0.030
30–34	3.00	(2.35–3.83)	<0.001	1.50	(1.08–2.09)	0.016	1.46	(1.05–2.04)	0.025	1.71	(1.06–2.77)	0.029	2.12	(1.03–4.39)	0.042	2.23	(1.08–4.61)	0.030
35 and above	1.00	Ref		1.00	Ref		1.00	Ref		0.82	(0.52–1.31)	0.415	1.32	(0.56–3.13)	0.539	1.40	(0.59–3.33)	0.441

^a^Multivariate aHR adjusted for all variables in the model. aHR, adjusted hazard ratio; CI, confidence interval.
