# Supplementary material for: Sexual partnership age pairings and risk of HIV acquisition in rural South Africa
Source: AIDS. 2017 Jul 12;31(12):1755–64. doi: 10.1097/QAD.0000000000001553 (PMC5508850; doi:10.1097/QAD.0000000000001553)
Supplement: Supplemental Digital Content [file aids-31-1755-s001.docx]

**Supplementary figure 1.** Participant selection flow-chart. *Mean (±SD) number of study visits was 2.2±1.4 for women and 1.9±1.1 for men, mean (±SD) observation time per exposure episode was 1.0±0.5 years for women and 0.9±0.5 years for men, and mean (±SD) age was 28.0±9.5 years for women and 26.2±9.4 years for men.

Completed at least one general health survey between 2004-2015

**N = 66,566 individuals**

Included in HIV cohort (tested HIV negative followed by at least one HIV test) **N = 18,285 individuals**

Not included in HIV cohort (never tested, HIV positive at first test, or HIV negative at first test without subsequent test) **N = 48,281 individuals**

**Partner-age sample*:** Non-missing information on most recent partner age on at least one follow-up visit and partner age ≥ 15 years

**N = 12,224 individuals**

< 15 years of age or > 49 years of age (women) or > 55 years of age (men) **N = 9**

Women (15-49 years of age) Men (15-55 years of age) **N = 18,276 individuals**

Missing information on partner age or partner age < 15 years of age on all survey visits **N = 5,875 individuals**

**Full Sample:**

**N = 18,099 individuals**

Survey visit more than one year before observation start or one year after observation end **N = 176 individuals**

**Supplementary table 1.** Age and gender-stratified, multivariate adjusted (by respondent age and calendar year) hazard ratios for the association between age of most recent sexual partner and risk of HIV.

|  | Women | | | | | |  | Men | | | | | | | | |
| --- | --- | --- | --- | --- | --- | --- | --- | --- | --- | --- | --- | --- | --- | --- | --- | --- |
|  | 15-24 |  |  | 25-49 |  |  |  | 15-24 |  |  | 25-34 |  |  | 35-55 |  |  |
|  | aHR | 95% CI | P-value | aHR | 95% CI | P-value |  | aHR | 95% CI | P-value | aHR | 95% CI | P-value | aHR | 95% CI | P-value |
| Age of respondent (years) | 0.99 | (0.95-1.04) | 0.695 | 0.95 | (0.92-0.97) | <0.001 |  | 1.19 | (1.08-1.30) | <0.001 | 0.97 | (0.89-1.07) | 0.563 | 1.01 | (0.94-1.08) | 0.816 |
| Age of most recent partner |  |  |  |  |  |  |  |  |  |  |  |  |  |  |  |  |
| 15-19 | 0.86 | (0.44-1.69) | 0.656 | . | . | . |  | 1.00 | ref |  | 1.00 | ref |  | 1.00 | ref |  |
| 20-24 | 0.87 | (0.47-1.61) | 0.657 | 0.71 | (0.10-5.18) | 0.732 |  | 0.97 | (0.65-1.46) | 0.890 | 5.24 | (0.72-38.28) | 0.102 | . | . | . |
| 25-29 | 1.02 | (0.55-1.87) | 0.956 | 1.73 | (1.14-2.63) | **0.010** |  | 1.00 | (0.30-3.32) | 0.994 | 5.87 | (0.8-43.29) | 0.082 | . | . | . |
| 30-34 | 1.19 | (0.61-2.31) | 0.614 | 1.64 | (1.14-2.36) | **0.008** |  | 11.58 | (1.53-87.92) | **0.018** | 7.88 | (0.98-63.1) | 0.052 | . | . | . |
| 35+ | 1.00 | ref |  | 1.00 | ref |  |  | . | . | . | . | . | . | . | . | . |

“.” Indicates not applicable or insufficient sample size
